# Prognostic Impact and Functional Annotations of KIF11 and KIF14 Expression in Patients with Colorectal Cancer

**DOI:** 10.3390/ijms22189732

**Published:** 2021-09-08

**Authors:** Izabela Neska-Długosz, Karolina Buchholz, Justyna Durślewicz, Maciej Gagat, Dariusz Grzanka, Krzysztof Tojek, Anna Klimaszewska-Wiśniewska

**Affiliations:** 1 Department of Clinical Pathomorphology, Faculty of Medicine, Collegium Medicum in Bydgoszcz, Nicolaus Copernicus University in Torun, 85-094 Bydgoszcz, Poland; iznes@cm.umk.pl (I.N.-D.); karolina.buchholz@cm.umk.pl (K.B.); justyna.durslewicz@cm.umk.pl (J.D.); d_grzanka@cm.umk.pl (D.G.); 2 Department of Histology and Embryology, Faculty of Medicine, Collegium Medicum in Bydgoszcz, Nicolaus Copernicus University in Torun, 85-092 Bydgoszcz, Poland; mgagat@cm.umk.pl; 3 Department of General, Colorectal and Oncological Surgery, Faculty of Medicine, Collegium Medicum in Bydgoszcz, Nicolaus Copernicus University in Torun, 85-168 Bydgoszcz, Poland; krzysztoftojek@cm.umk.pl

**Keywords:** colorectal cancer, KIF11, KIF14, prognostic factor, genomic instability

## Abstract

Genomic instability (GIN) has an important contribution to the pathology of colorectal cancer (CRC). Therefore, we selected mitosis and cytokinesis kinesins, KIF11 and KIF14, as factors of potential clinical and functional value in CRC, as their aberrant expression has been suspected to underlie GIN. We examined the expression and the prognostic and biological significance of KIF11 and KIF14 in CRC via in-house immunohistochemistry on tissue microarrays, public mRNA expression datasets, as well as bioinformatics tools. We found that KIF11 and KIF14 expression, at both the protein and mRNA level, was markedly altered in cancer tissues compared to respective controls, which was reflected in the clinical outcome of CRC patients. Specifically, we provide the first evidence that KIF11 protein and mRNA, *KIF14* mRNA, as well as both proteins together, can significantly discriminate between CRC patients with better and worse overall survival independently of other relevant clinical risk factors. The negative prognostic factors for OS were high KIF11 protein, high KIF11 protein + low KIF14 protein, low *KIF11* mRNA and low *KIF14* mRNA. Functional enrichment analysis revealed that the gene sets related to the cell cycle, DNA replication, DNA repair and recombination, among others, were positively associated with *KIF11* or *KIF14* expression in CRC tissues. In TCGA cohort, the positive correlations between several measures related to GIN and the expression of KIFs were also demonstrated. In conclusion, our results suggest that CRC patients can be stratified into distinct risk categories by biological and molecular determinants, such as KIF11 and KIF14 expression and, mechanistically, this is likely attributable to their role in maintaining genome integrity.

## 1. Introduction

Colorectal cancer (CRC) is the third most commonly diagnosed malignancy and simultaneously the second leading cause of cancer death globally [1]. Its incidence depends on many variables, among which the most relevant are behavioral factors (including obesity, sedentary lifestyle, high intake of red meat, smoking and alcohol consumption) and loss of genomic stability leading to the accumulation of genetic and/or epigenetic alterations and consequently to the development of invasive cancer [2]. Several genomic instability (GIN)-associated genes presenting a significant contribution to CRC progression have been identified so far [3]. These findings resulted in improving the diagnosis process and devising effective personalized therapies that enabled some CRC patients to obtain clinical benefits [4,5,6]. Nevertheless, because CRC is a heterogeneous disease manifested by various driving mutations, there is a need for further research to find novel biomarkers and therapeutic targets. Therefore, based on the knowledge that tumorigenesis may be accompanied by genetic alterations leading to GIN, we selected kinesin family member 11 (KIF11) and kinesin family member 14 (KIF14) involved in the cell division as potential biomarkers of CRC, the impaired expression of which may be associated with CRC pathogenesis and/or shorter survival time of CRC patients.

Due to the above, the first stage of the present study was the immunohistochemical evaluation of KIF11 and KIF14 expression and distribution both in colorectal cancer specimens and nontumor adjacent tissues. The obtained protein expression data were used to assess the protein correlation, as well as to examine the expression levels with regards to clinicopathological variables and overall survival (OS) of CRC patients. The same analyses were performed based on the gene expression data sourced from The Cancer Genome Atlas (TCGA) and Genotype-Tissue Expression (GTEx) databases for colorectal cancer tumors and normal colon mucosa samples, respectively. Finally, a functional enrichment analysis based on the top 50 genes positively correlated with *KIF11* or *KIF14* was performed to predict biological functions and pathways related to KIF11 or KIF14 in CRC.

## 2. Results

### 2.1. Immunohistochemical Expression of KIF11 and KIF14 Proteins: Association with Clinicopathological Parameters

**Figure 1:**
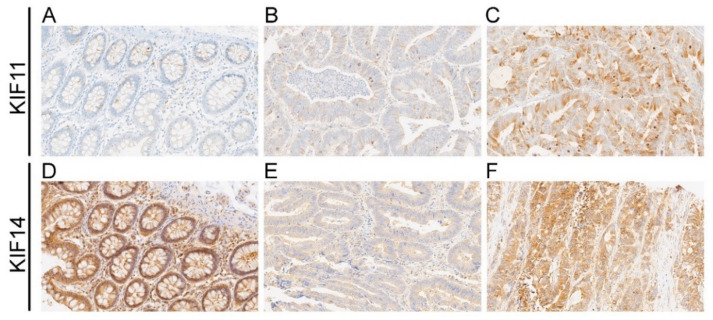
Representative photographs showing immunohistochemical expression of KIF11 (**A**–**C**) and KIF14 (**D**–**F**) in colorectal cancer (CRC) and adjacent tissue (control). KIF11 staining in control tissue (**A**); weak staining (**B**) and strong staining (**C**) for KIF11 in CRC; KIF14 staining in control tissue (**D**); weak staining (**E**) and strong staining (**F**) for KIF11 in CRC. Original magnification 20×.

**Figure 2:**
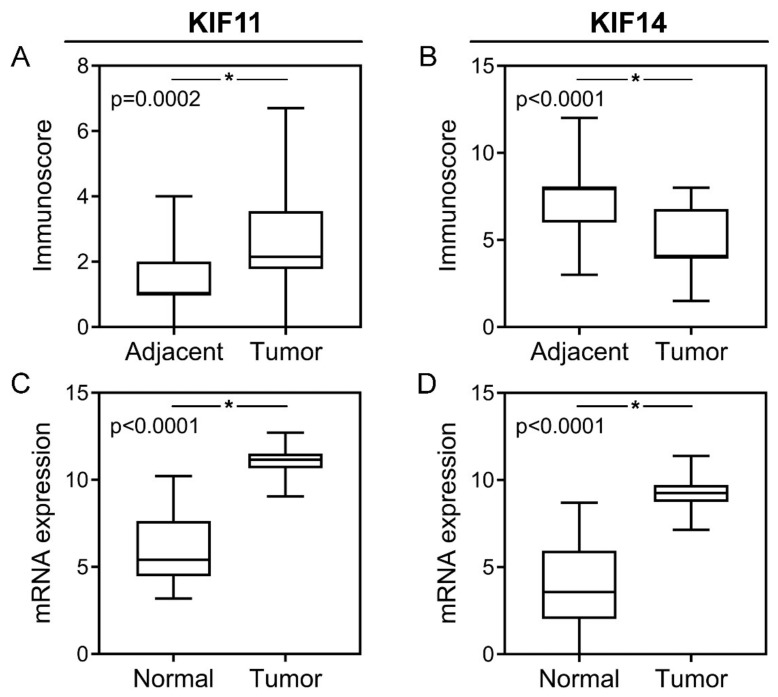
Protein and mRNA expression of KIF11 and KIF14 in colorectal cancer. KIF11 (**A**) and KIF14 (**B**) protein expression levels in CRC tumors compared to noncancerous adjacent tissues; *KIF11* (**C**) and *KIF14* (**D**) mRNA expression levels in CRC tumors compared to normal tissues. The error bars present the range from minimum to maximum values of data. Asterisks (*) indicate statistically significant differences (*p* < 0.05).

KIF11 labeling was cytoplasmic with occasional concurrent staining of the membrane (n = 4/86; 4.65%) in cancer cells and fully restricted to the cytoplasm in nontumor cells (Figure 1A–C). In the case of KIF14, tumor tissues demonstrated cytoplasmic labeling but accompanied by nuclear or membranous staining in some CRC samples (n = 28/86, 32.56% and n = 12/86, 13.95%, respectively). In nontumor tissues, it was primarily membranous-cytoplasmic immunoreactivity with simultaneous nuclear staining in several cases (n = 9/24; 37.5%; Figure 1D–F). Based on cut-off values established for KIF11 and KIF14, the positive expression of these proteins was found in 25 (29.07%) and 29 (33.72%) CRC cases, while regarding the control group, it was 4 (16.67%) and 16 (66.67%) cases, respectively. According to the above data, the expression level of KIF11 was significantly up-regulated in CRC tissues in comparison to the adjacent noncancerous tissues (*p* = 0.0002; Figure 2A) and down-regulated in the case of KIF14 (*p* < 0.001; Figure 2B).

The level of KIF11 expression did not demonstrate a statistically significant relationship with any examined clinicopathological features of CRC patients (*p* > 0.05; Table 1). In turn, the aberrant expression of KIF14 was markedly associated with vascular invasion (VI) in CRC samples (*p* = 0.01; Table 1). Apart from that, no relationships between KIF14 expression status and the remaining clinicopathological data, such as age, gender, grading, perineural invasion (PNI) as well as pT, pN and pM status were found (*p* > 0.05; Table 1). Furthermore, there was no significant correlation between KIF11 and KIF14 expression levels (r = 0.179; *p* = 0.099).

### 2.2. Immunohistochemical Expression of KIF11 and KIF14 Proteins: Association with Overall Survival

**Figure 3:**
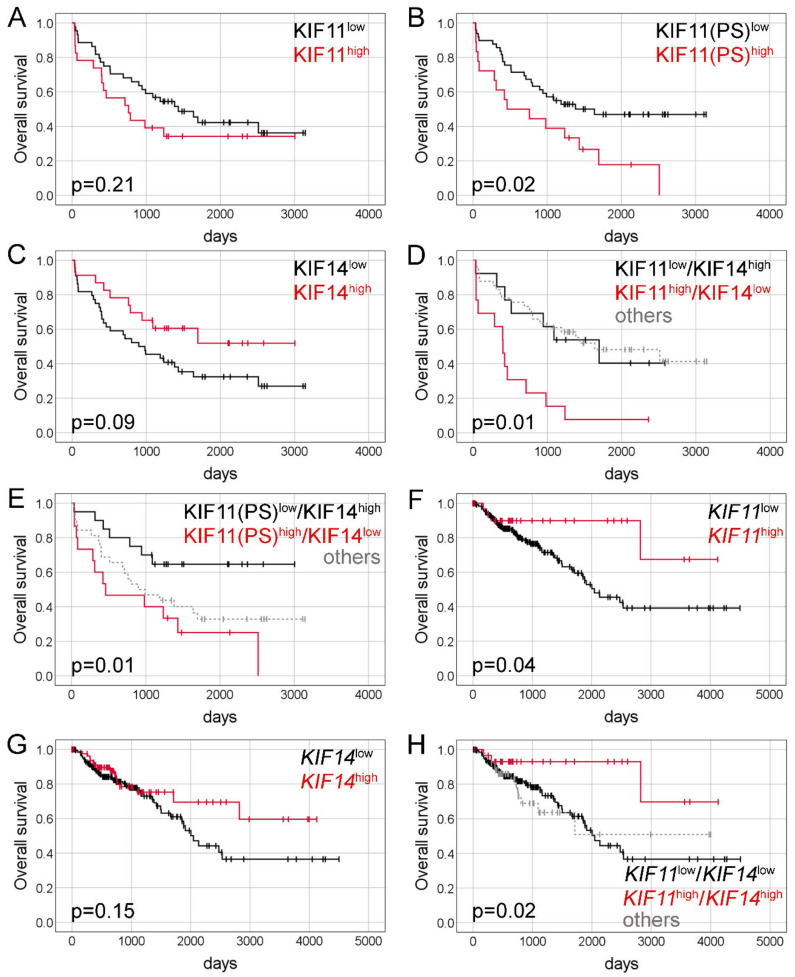
Kaplan-Meier curves presenting the overall survival of CRC patients depending on KIF11 protein expression (**A**); KIF11 protein expression assessed based on PS values (**B**); KIF14 protein expression (**C**); combined KIF11 (IRS)/KIF14 protein expression (**D**); combined KIF11(PS)/KIF14 protein expression (**E**); *KIF11* mRNA expression (**F**); *KIF14* mRNA expression (**G**) and combined *KIF11*/*KIF14* mRNA expression (**H**).

Kaplan-Meier survival analysis indicated that KIF11 expression by IRS score was not associated with OS time of CRC patients (*p* = 0.21; Figure 3A). The median survival time for the patients with a high and low level of KIF11 expression was 760 days and 1432 days, respectively. However, when PS (cut-point = 2.3) instead of IRS was taken as a measure of KIF11 expression, the same analysis demonstrated that CRC patients with KIF11 overexpression (n = 23) had markedly shorter OS time than those with KIF11 underexpression (459 days vs. 1637 days, *p* = 0.02; Figure 3B). In the case of KIF14, Kaplan-Meier estimation revealed that high expression of this protein was related to better OS, although this was not a significant association (not reached vs. 899 days, *p* = 0.09, Figure 3C). When both these markers were evaluated together, CRC patients who coexpressed KIF11 (IRS) at high level and KIF14 at low level had markedly shorter OS than those with the opposite expression pattern (400 days vs. 1696 days; *p* = 0.01; Figure 3D); a similar relationship was found when PS was used to measure KIF11 expression (459 days vs. not reached; *p* = 0.01; Figure 3E).

Univariate Cox analysis identified KIF11 PS (HR = 2.17, 95% CI 1.14–4.12; *p* = 0.02), KIF11(IRS)^high^/KIF14^low^ (HR = 3.33, 95% CI 1.29–8.55; *p* = 0.01), KIF11(PS)^high^/KIF14^low^ (HR = 3.29, 95% CI 1.29–8.37; *p* = 0.01), and pM status (HR = 3.27, 95% CI 1.64–6.53; *p* = 0.001) as significant prognostic variables for OS (Table 2). KIF14 expression (HR = 0.54, 95% CI 0.26–1.10; *p* = 0.09) trended towards a correlation with OS (Table 2). When examined in multivariate Cox analysis, KIF11 PS (adjusted HR = 2.41, 95% CI 1.10–5.28; *p* = 0.03), KIF11(IRS)^high^/KIF14^low^ (adjusted HR = 3.91, 95% CI 1.13–13.54; *p* = 0.03), KIF11(PS)^high^/KIF14^low^ (adjusted HR = 5.72, 95% CI 1.74–18.83; *p* = 0.004), and pM status (adjusted HR = 3.09, 95% CI 1.37–6.99; *p* = 0.007) persisted as independent prognostic factors for OS. Notably, as compared with established prognostic markers, as well as single expression of either proteins, combined KIF11 and KIF14 expression had the highest prognostic hazard ratio values in the multivariate analysis (Table 2). After correction for bias caused by the univariate analysis, tumor grade predicted OS independently of age at diagnosis, gender, pT, pN, pM and the study (adjusted HR = 3.62, 95% CI 1.14–11.53; *p* = 0.03; Table 2).

### 2.3. Expression of KIF11 and KIF14 Genes: Association with Clinicopathological Parameters

In silico analysis demonstrated that *KIF11* and *KIF14* expression levels were significantly up-regulated in CRC tumors in comparison to normal tissue samples (*p* < 0.0001 for both; Figure 2C,D), and their overexpression was observed in 33 (11.91%) and 83 (29.96%) of CRC cases, respectively. *KIF11* expression was not significantly associated with any examined clinicopathological features (*p* > 0.05; Table 3). In turn, elevated *KIF14* levels were more frequently detected in CRC patients with lymph node metastases (*p* = 0.045) than in those without cancer cells in lymph nodes (36.52% vs. 25.00%). Moreover, the prevalence of KIF14 overexpression was higher in stage III-IV tumors compared to I-II ones (38.14% vs. 23.33%; *p* = 0.01). No other relationships between the expression level of *KIF14* and examined clinicopathological features, including age, gender, pT and pM status were found (*p* > 0.05; Table 3).

*KIF11* and *KIF14* expression levels were significantly correlated with each other (r = 0.76; *p* < 0.0001). There were also significant correlations between *KIF11* and MSI MANTIS score (r = 0.18; *p* = 0.003), MSIsensor score (r = 0.19; *p* = 0.002), and mutation count (r = 0.22; *p* = 0.0003), whereas *KIF14* was significantly associated with mutation count (r = 0.14; *p* = 0.02), marginally significantly with MSIsensor score (r = 0.12; *p* = 0.05), but not with MSI MANTIS score (r = 0.08; *p* = 0.19). Both *KIF11* and *KIF14* were significantly associated with *MSH6* (r = 0.55; *p* < 0.0001; r = 0.57; *p* < 0.0001, respectively), *MSH2* (r = 0.57; *p* < 0.0001; r = 0.50; *p* < 0.0001, respectively), *MLH1* (r = 0.20; *p* = 0.001; r = 0.19; *p* = 0.002, respectively), and *PMS2* (r = 0.20; *p* = 0.001; r = 0.32; *p* < 0.0001, respectively).

### 2.4. Expression of KIF11 and KIF14 Genes: Association with Overall Survival

Kaplan-Meier survival analysis demonstrated that high *KIF11* expression was noticeably associated with better OS of CRC patients (not reached vs. 2047 days; *p* = 0.04; Figure 3F). Likewise, patients with overexpression of *KIF14* tended to survive longer than those with its underexpression (not reached vs. 2047 days), although the survival difference was statistically insignificant (*p* = 0.15; Figure 3G). Moreover, CRC patients with simultaneous high expression of *KIF11* and *KIF14* had markedly better OS than those with low-level expression of both genes (not reached vs. 2047 days; *p* = 0.02; Figure 3H).

In the univariate Cox analysis, *KIF11* (HR = 0.36, 95% CI 0.13–0.996; *p* = 0.049), combined *KIF11*/*KIF14* expression (HR = 0.27, 95% CI 0.08-0.88; *p* = 0.03), tumor stage (HR = 2.60, 95% CI 1.55–4.35; *p* = 0.001) as well as pT (HR = 3.21, 95% CI 1.17–8.84; *p* = 0.02), pN (HR = 2.46, 95% CI 1.50–4.04; *p* < 0.0001), and pM status (HR = 4.21, 95% CI 2.34–7.56; *p* < 0.0001) were significantly correlated with OS (Table 4). Multivariate Cox proportional hazards models validated high *KIF11* (adjusted HR = 0.32, 95% CI 0.11–0.89; *p* = 0.03) and *KIF11*+*KIF14* (adjusted HR = 0.22, 95% CI 0.07–0.71; *p* = 0.01) as positive, and advanced tumor stage as negative, markers for OS (Table 4). Importantly, after correction for bias caused by the univariate analysis, elevated *KIF14* expression (adjusted HR = 0.47, 95% CI 0.26–0.86; *p* = 0.02), as well as higher age, appeared as an independent positive or negative prognostic factor for OS, respectively (Table 4).

### 2.5. Expression of KIF11 and KIF14 Genes: Functional Enrichment Analysis

The top 50 genes that were positively correlated (upregulated DEGs; uDEGs) with *KIF11* or *KIF14* in colon cancer tissues were determined using TCGA dataset and the UALCAN web-based tool. Centrosomal Protein 55 (*CEP55*) and Rho GTPase activating protein 11A (*ARHGAP11A*) had the highest positive correlation with *KIF11* (Spearman’s correlation coefficient r ≥ 0.80), whereas abnormal spindle-like microcephaly-associated protein (*ASPM*) and centromere protein F (*CENPF*) were the top positively correlated genes with *KIF14* (r ≥ 0.85). Both KIFs were also strongly correlated with *MKI67* (r ≥ 0.72). Correlation analysis of CRC patients enrolled from TCGA via the UCSC Xena database confirmed these correlations (*KIF11* and *CEP55*: r = 0.84; *p* < 0.0001; *KIF11* and *ARHGAP11A*: r = 0.77; *p* < 0.0001; *KIF14* and *ASPM*: r = 0.90; *p* < 0.0001; *KIF14* and *CENPF*: r = 0.89; *p* < 0.0001; *KIF11* and *MKI67*: r = 0.78; *p* < 0.0001; *KIF14* and *MKI67*: r = 0.74; *p* < 0.0001).

**Figure 4:**
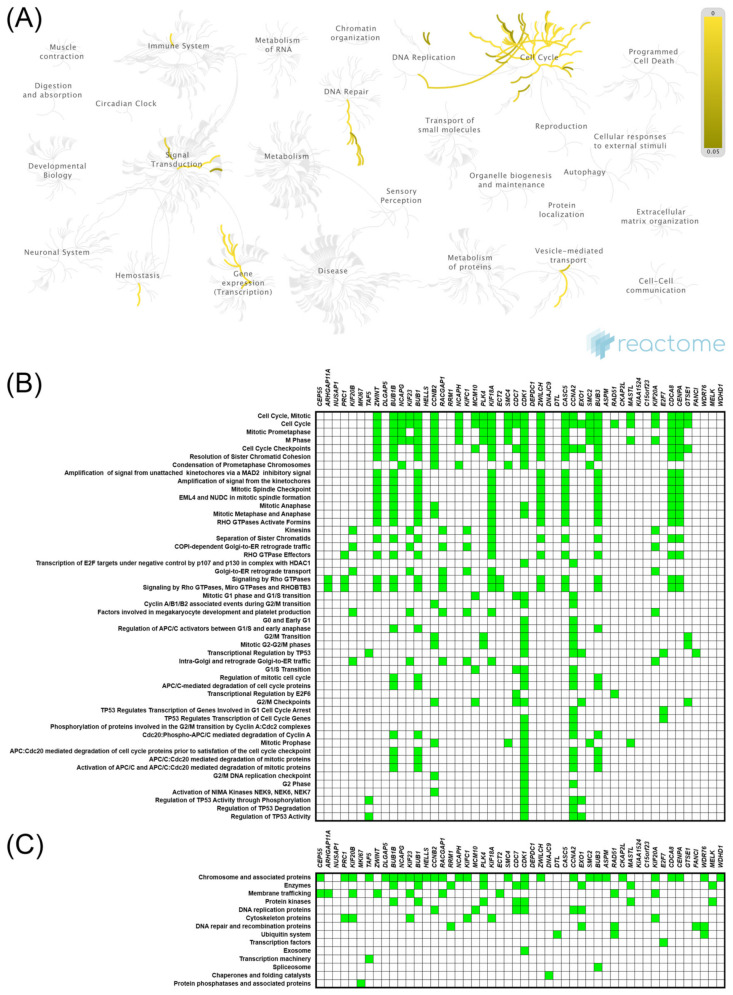
Functional enrichment analysis based on the TCGA dataset and UALCAN web tool. The top 50 genes and reactome pathways positively correlated with *KIF11* expression (**A**,**B**); BRITE functional hierarchies for the top 50 genes that were co-upregulated with *KIF11* (**C**).

**Figure 5:**
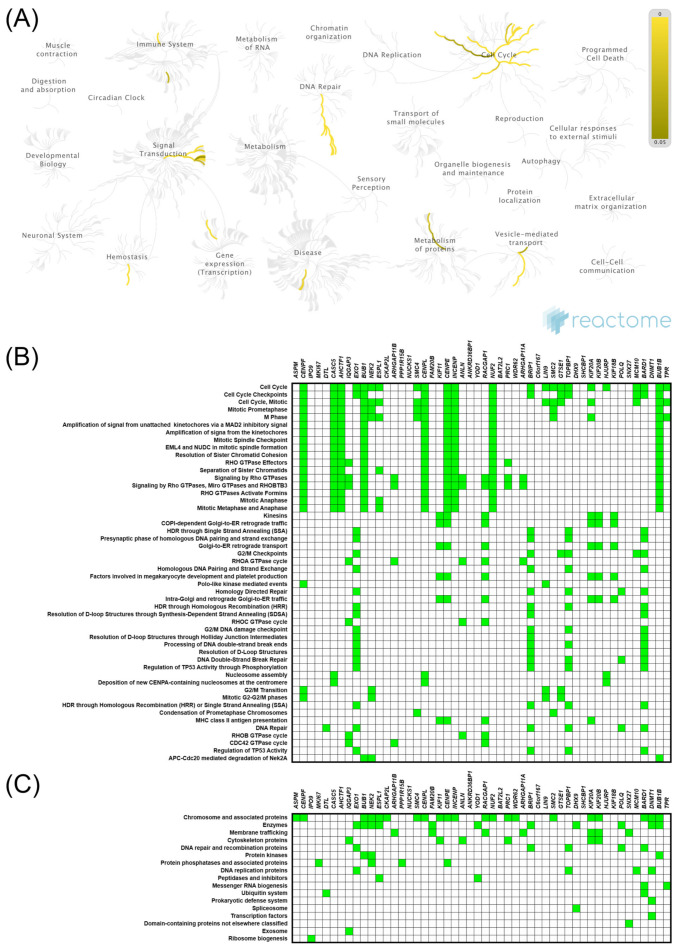
Functional enrichment analysis based on the TCGA dataset and UALCAN web tool. The top 50 genes and reactome pathways positively correlated with *KIF14* expression (**A**,**B**); BRITE functional hierarchies for the top 50 genes that were co-upregulated with *KIF14* (**C**).

uDEGs were inputted into the STRING and Cytoscape, where the PPI networks were constructed, in which 50 nodes correlating with either *KIF11* (Figure S1) or *KIF14* (Figure S2) formed the networks with 937 or 454 edges, respectively (PPI enrichment *p* values < 1.0 × 10^−16^; local clustering coefficient 0.92 and 0.72). The hub genes in the PPI networks were obtained with the Cytoscape plugin cytoHubba, taking degree as a node ranking method. The top 10 hub genes in the *KIF11*-correlated network are displayed in Figure S1, of which *CDK1* and *BUB1* have the highest scores. Figure S2 comprises similar analysis for *KIF14*, whereby it is *CENPE* and *BUB1B* that are the hub genes in this network. Furthermore, we performed Reactome and KEGG BRITE enrichment analyses to predict the putative functions of *KIF11* or *KIF14*. The Reactome Pathway hierarchy panels for the top 50 genes coexpressed with *KIF11* or *KIF14* are illustrated in Figure 4A and Figure 5A, respectively. Reactome pathway analysis for *KIF11* showed that the co-upregulated genes were mainly involved in “cell cycle, mitotic”, “mitotic prometaphase”, “cell cycle checkpoints”, “resolution of sister chromatid cohesion”, “condensation of prometaphase chromosomes”, and “amplification of signal from the kinetochores” (Figure 4A,B). KEGG BRITE functional hierarchies for *KIF11* showed that the coupregulated genes had the preponderance of genes representing “chromosome and associated proteins”, “enzymes”, “membrane trafficking proteins”, “protein kinases”, “DNA replication proteins”, “cytoskeleton proteins”, and “DNA repair and recombination proteins”, among others (Figure 4C). Similar results were obtained for *KIF14*, and details of these analyses are depicted in Figure 5.

**Figure 6:**
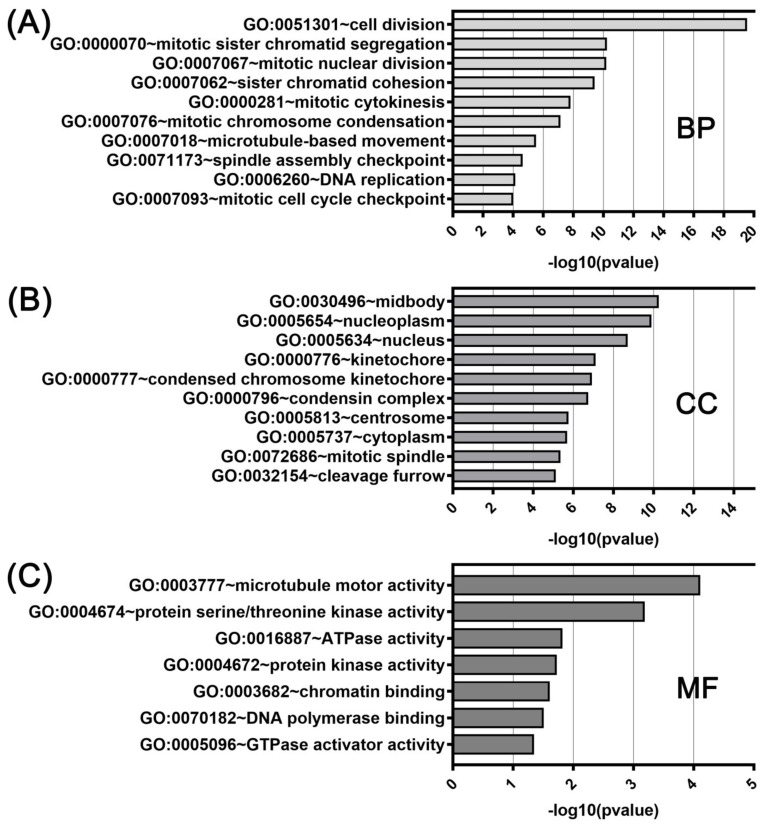
DAVID functional GO analysis of biological processes (**A**; BP), cellular components (**B**; CC), and molecular functions (**C**; MF). The top 10 GO terms for BP, CC and 7 GO terms for MF are presented for genes co-upregulated with *KIF11*. The *p* value was calculated and sorted with −log10(*p* value).

**Figure 7:**
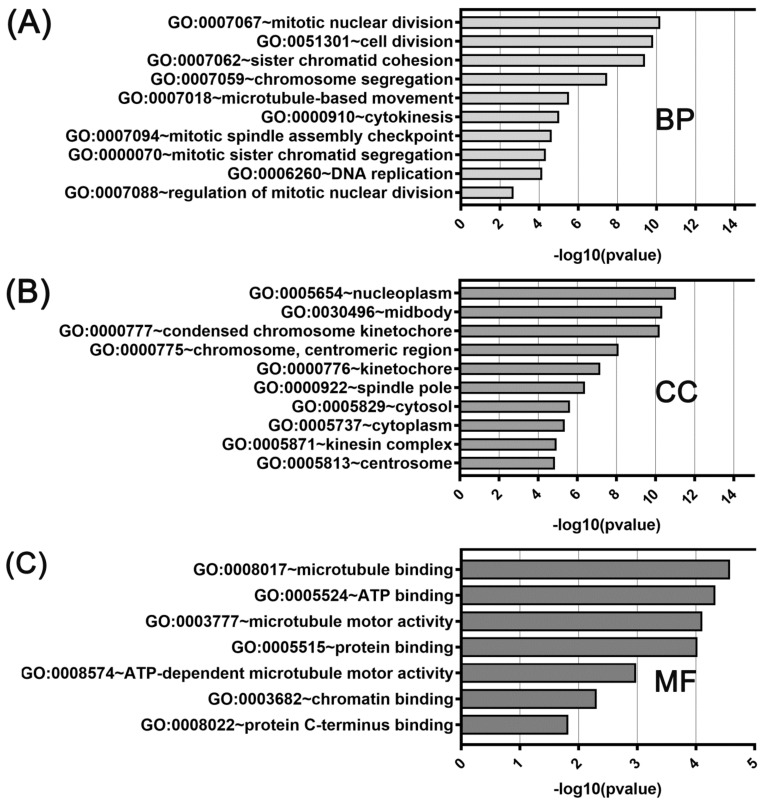
DAVID functional GO analysis of (**A**) biological processes (BP), (**B**) cellular components (CC), and (**C**) molecular functions (MF). The top 10 GO terms for BP, CC and 7 GO terms for MF are presented for genes co-upregulated with *KIF14*. The *p* value was calculated and sorted with −log10(*p* value).

GO function enrichment was performed with genes coexpressed with either *KIF11* or *KIF14* using the DAVID tool to analyze their possible activities in biological processes, molecular functions, and cellular components. In GO analysis for *KIF11* and its coexpressed genes, the most enriched ontology terms were GO:0051301 (cell division; Figure 6A), GO:0030496 (midbody; Figure 6B), and GO:0003777 (microtubule motor activity; Figure 6C). Likewise, the enriched functional GO terms related to the *KIF14*-gene network included GO:0007067 (mitotic nuclear division; Figure 7A), GO:0005654 (nucleoplasm; Figure 7B), and GO:0008017 (microtubule binding; Figure 7C).

## 3. Discussion

Over the last few years, numerous studies aiming to identify novel biomarkers involved in colorectal cancer pathogenesis and progression have been carried out. Findings in this field focused scientists’ attention on genome instability, especially microsatellite and chromosome instability, as a crucial driving force in colorectal tumorigenesis, and consequently allowed establishment of several diagnostically and clinically valuable CRC biomarkers [3]. We considered that this research direction is justifiable and requires continuation. Therefore, based on the scientific literature [7,8,9,10,11], we chose mitosis and cytokinesis proteins, KIF11 and KIF14, as factors of potential diagnostic and prognostic value in CRC, since their aberrant expression has been suspected to underlie GIN. At first, we assessed the immunohistochemical expression of proteins in the context of selected clinicopathological traits and the overall survival of CRC patients. Thereafter, the same analyses were performed using *KIF11* and *KIF14* mRNA expression data retrieved from public sources. Finally, the genes coexpressed with *KIF11* or *KIF14* in colon adenocarcinoma were identified and functionally annotated.

KIF11 (also known as EG5) is a motor protein belonging to the kinesin-like protein family. It is responsible for spindle dynamics, as it takes part in chromosome positioning, centrosome separation, and bipolar spindle formation during mitosis [12,13]. KIF11 expression was found to be altered and associated with patient survival in numerous types of human cancer, which suggests its contribution to cancer development and progression [14,15,16,17,18]. Nevertheless, to our best knowledge, apart from the current report, only one research to date has presented a clinical value of KIF11 protein for survival stratification of CRC patients [19]. In that study, Imai et al. evaluated the immunoexpression of KIF11 in CRC tissues taking into account percentage score and, based on this, KIF11-positivity was reported to be more than twice as high compared to percentage positivity obtained by us (62% vs. 27%). However, they set the cut-off point at 10% of stained tumor cells, while our positivity threshold was set higher, at no less than 25%. Nevertheless, it should be emphasized that as the primary readout we decided to use the IRS score, which incorporates both intensity and proportion. This was because various staining intensities are frequently observed in clinical practice, and these may have a biological meaning. Indeed, our recent pancreatic cancer data on KIF11 has revealed that the IRS scoring system was more informative for prognosis than PS itself [20]. Importantly, in our studies, we avoided choosing arbitrary cut-off values for the interpretation of target protein expression, and therefore we adopted outcome data-derived method from the Evaluate Cutpoint software [21]. Nevertheless, we agree that the evaluation of immunoexpression in terms of the proportion of the positive staining cells seems adequate in CRC tissues, as in our series it was PS score and not IRS score that was significantly associated with patient prognosis. Specifically, we found that high KIF11 expression via proportion score may serve as a potential predictor of dismal survival rates in CRC, independently of age at diagnosis, gender, grade, and T, N, and M stages. This finding was in contrast to the study of Imai et al., who did not see any significant impact of KIF11 expression on the survival of CRC patients [19]. However, it fits well with the functional portrait of KIF11 in most tumor types studied to date [15,16,17,18,22]. Moreover, we did not find any correlations of either IRS score or PS score (Supplementary Table S1) with clinicopathological parameters of CRC patients, which further supports the independence of KIF11 expression from potential confounders. Contrary to our study, Imai et al. demonstrated a significant association of KIF11 expression via PS score with pT status and degree of cancer differentiation. Moreover, they showed that KIF11 overexpression was an early event in CRC pathogenesis, as it was detected both in low-grade and high-grade tumors with a similar high frequency. Our cohort of patients was characterized by a specific distribution of the clinicopathological data. It did not contain pT1 and G1 tumors, which was a limitation of the current study and, simultaneously, the probable reason why we could not verify correlations presented by Imai et al., as well as another explanation for the lower positivity rate of KIF11 expression among our cases. Finally, a distinct KIF11 staining pattern was observed between our CRC cohort and that of Imai et al. [19]. In our hands, the staining pattern for KIF11 was predominantly cytoplasmic, whereas Imai and coworkers observed mainly nuclear staining of CRC cells, likely due to the use of different antibodies. In turn, in agreement with our results, the cited authors showed that the mRNA levels of *KIF11* were significantly upregulated in CRC tissues versus normal tissues. Similar results were reported by Zhou et al., who also revealed the clinicopathological relevance of *KIF11* mRNA in CRC by showing that its high expression was intimately correlated with clinical parameters, such as T stage, TNM stage, Ki-67 status and vessel invasion [23]. As far as we are aware, the current report is, however, the first to present prognostic significance of *KIF11* mRNA in CRC. Although we observed the same expression status for KIF11 protein and mRNA in CRC tissues (upregulation for both), they carried the opposite prognostic significance. Indeed, in contrast to KIF11 protein, high *KIF11* mRNA was associated with noticeably better OS and constituted an independent predictor of improved OS in the TCGA cohort. This finding contradicts the reported poor prognosis of ovarian, pancreatic, breast or non-small cell lung cancer patients overexpressing *KIF11* mRNA [20,24,25,26]. Nevertheless, given that protein levels equate more closely with function than mRNA levels [27], it is likely that elevated KIF11 expression is functionally linked to an adverse prognosis in CRC. In support of this notion, Imai et al. showed that knockdown of *KIF11* in CRC and other gastrointestinal (GI) cancer cell lines significantly reduced the number and size of spheres formed by analyzed cells [19,28]. In the light of this and our protein data, it seems that KIF11 may produce a novel molecular target for colorectal cancer therapy.

KIF14 is a microtubule motor protein belonging to the kinesin-3 family that is known to play an essential role in cytokinesis during cell division through the internal motor domain with microtubule-dependent ATPase activity, but it is also involved in other biological processes such as proliferation, intracellular transport, and apoptosis [29,30,31]. KIF14 is called an oncogenic kinesin, and most reports have shown that it is overexpressed in numerous human cancers and correlated with a poor prognosis [32,33,34,35]. However, there are also studies implying the tumor-suppressive function of KIF14 in some tumors [20,36]; thus, its precise role may be tumor and/or context-dependent. Despite its importance, as far as we are aware, the current study is the first to investigate the clinical value of KIF14 protein in CRC patients. According to our results, and unlike the data published for other examined cancers, KIF14 protein expression was downregulated in CRC tissues compared to the adjacent noncancerous tissues. In turn, *KIF14* mRNA expression was upregulated in tumors compared to normal tissues, which is in line with the previous reports on various cancers, including colorectal cancer [37,38,39,40,41,42]. A mismatch between mRNA and protein levels has been frequently observed and extensively debated in the literature [43,44]. It is widely accepted that mRNA expression correlates too weakly with protein expression for it to be a reliable predictor of protein expression [45], supporting the importance of profiling mRNA levels in parallel to protein abundance and degradation rates [46]. This has been particularly demonstrated with transcription factors, signaling genes, chromatin modifying genes, and genes with cell-cycle-specific functions, which are known to have unstable mRNA and unstable protein [43]. Consistent with its primary role in cell division, KIF14 would be expected to have a negative correlation between mRNA and protein. Furthermore, even though CRC has been shown to be enriched for negative correlations between protein and mRNA expression patterns [47], there is still a need to validate whether indeed protein and mRNA levels of KIF14 are discordantly expressed in CRC tissues, whereby both measurements should be performed simultaneously in the same cases.

Notably, in our Kaplan-Meier analysis, a certain trend towards the association between *KIF14* status and OS in patients with CRC was revealed. In the multivariate Cox model, accounting for additional covariates, the strength of the association increased and was statistically significant. Thus, the present investigation identified *KIF14* expression as an independent favorable prognostic factor, although it was not reflected in the other report demonstrating that *KIF14* gene expression did not affect the survival of CRC patients [42]. Contrary to the TCGA cohort used in the present study, the cited research was carried out on a relatively small cohort group of patients with little ethnic diversity, and cut-off values for high and low gene expression were established using different methods, which may explain the divergent results. In our study, a suggestive association between KIF14 expression and better overall survival of CRC patients was found also at the protein level. The current study may simply have been underpowered to observe a significant impact of KIF14 protein, because of the relatively small number of cases in our cohort. Therefore, our results await replication in future studies with expanded sample sizes.

Having established clinical significance of KIF11 and KIF14 in CRC, we performed the Reactome Pathway, GO terms enrichment, and KEGG BRITE of genes coexpressed with them to predict the biological processes of the KIFs in the pathology of these tumors. Recent experimental research has suggested that the aberrant expression of KIF11 (either upregulation [48] or silencing [23,49]), may be a pathogenic event contributing to cancer development and/or progression through genomic instability. *KIF11* was also identified as chromosome instability (CIN) gene in HCT116 colorectal cancer cell line [49]. Hence, it is not unexpected that the gene sets related to the cell cycle, DNA replication, DNA repair and recombination, among others, were positively associated with *KIF11* expression in CRC tissues of the TCGA cohort, since dysregulation of these processes is considered the main sources of GIN [50,51]. Using this resource, we explored several measures related to genomic instability, including the MSI MANTIS score [52], MSIsensor score [53], and mutation count, and found their positive correlations with *KIF11* expression. Furthermore, *KIF11* was positively associated with the expression of mismatch repair (MMR) genes—*MSH6*, *MSH2*, *MLH1*, and *PMS2*, as well as the *MKI67* gene (coding for proliferation marker Ki-67), which is in agreement with the observations that development of GIN is paralleled by the upregulation of MMR genes and overlaps with the proliferative activity of tumor cells [54,55,56]. Given that mRNA levels of *KIF11* and *KIF14* were positively and strongly correlated with each other in CRC tissues, the functional and pathway analysis for *KIF14* allowed us to capture similar underlying biological processes as for *KIF11*, including the cell cycle; hence, the associated molecular components were e.g., chromosome-associated proteins. This seems consistent with the premise that GIN is possibly assisted by aberrant *KIF14* expression, which is supported by both experimental [30] and clinical (noncolorectal) [57,58] data. Therefore, we suggest that the marked differences in the survival outcome of CRC patients are at least partially based on genome instability, which occurs due to the failures of the genome stability pathways, of which KIF11 and KIF14 seem to be important players. Although GIN is a fundamental phenomenon of almost all human cancers, the exact role of these comprehensive processes at each stage of tumorigenesis is still obscure [59]. However, it is becoming clear that in some contexts, and depending on the level of genomic damage, GIN may either drive tumor progression or suppression [60]. Simultaneously, given that excessive CIN is lethal, tumors may select for alterations that antagonize the effects of excessive chromosome segregation errors, and increased genome stability can be selected to aid tumor growth [61]. Future works are, therefore, needed for a precise understanding of the molecular mechanisms underlying the prognostic effect of KIF11 and KIF14 in CRC.

This study has several limitations. The first is the source and nature of datasets, as our protein and mRNA data were not collected in single experiments on the same samples, and both datasets have a retrospective nature. The second is the lack of experimental verification. Even if the prognostic relevance of KIF11 and KIF14 for CRC was established, and underlying biological processes were predicted, the deep molecular mechanisms remained unknown. Moreover, missing values were present in our retrospective cohort study, which could reduce a statistical power of the study and the representativeness of the samples. Thus, further investigations are required to evaluate the model performance based on a larger cohort with complete data.

## 4. Materials and Methods

### 4.1. Tissue Material and Clinicopathological Data

The research was performed on formalin-fixed paraffin-embedded (FFPE) tissue samples including 86 CRC tumors and 24 adjacent noncancerous mucosa specimens collected from patients undergoing colectomy due to adenocarcinoma between 2010 to 2017, constituting the archival tissue collection of the Department of Clinical Pathomorphology, Collegium Medicum in Bydgoszcz, Nicolaus Copernicus University in Toruń (Poland). The clinical stages of all CRC tumors were reexamined according to the TNM 8th edition classification of the American Joint Committee on Cancer (AJCC) criteria [62]. A part of this cohort was previously described [63]. The study protocol was approved by the Bioethics Committee at Collegium Medicum in Bydgoszcz of Nicolaus Copernicus University in Toruń (no. 337/2018).

### 4.2. Survival Data

Of 86 CRC patients, 19 were excluded from survival analysis because of the lack of survival data or due to postoperative death occurring within 30 days after surgery. Finally, survival analysis was performed using survival data of 67 CRC patients. OS was defined as the time from resection until all-cause death or until the last follow-up date. The median OS time was 1188 days, while the median follow-up time was 2104 days.

### 4.3. Immunohistochemical Analysis

Tissue microarrays (TMAs) construction and then immunohistochemical staining of slides with tissuecores were performed as previously described [63]. For the majority of cases, four 2.0 mm cores were sampled from different tumor areas. For several cases (n = 7 and 6), a single 2-mm tissue core per donor tissue was used. Evaluation of KIF11 and KIF14 protein expression was carried out using the primary antibodies including rabbit polyclonal anti-KIF11 (1:500, 32 min; cat. no: PA5-82394, Thermo Fisher Scientific, Waltham, MA, USA) and rabbit polyclonal anti-KIF14 (1:150, 30 min; cat. no: HPA038061, Sigma-Aldrich, St. Louis, MO, USA), respectively. Thereafter, antigen-antibody complexes were visualized with the EnVision FLEX+ System (Dako, Agilent Technologies, Santa Clara, CA, USA) according to the manufacturer’s instructions. The semiquantitative assessment of the protein expression level was performed by two pathologists (IND, DG) at 20× original objective magnification. The staining score was based on the modified Remmele and Stegner scale (IRS; 0–12) [64] obtained by multiplying the percentage of positively stained cells/tissue area (PS; 0 < 5%; 1 = 5–24%; 2 = 25–49%, 3 = 50–74%; 4 ≥ 75%) and intensity of staining (IS; 0—negative, 1—weak, 2—moderate, 3—strong) for both KIF11 and KIF14. The cutoff points allowing segregation of immunoexpression levels of selected proteins into low and high were determined using the cutp function of the Evaluate Cutpoints application [21] and were 3.3 for KIF11 and 5.5 for KIF14. Scores below these values were interpreted as negative (low expression), whereas equal or above were defined as positive (high expression). Cases with the coexpression of KIF11^high^ and KIF14^low^ were analyzed against those with the opposite expression pattern (KIF11^low^/KIF14^high^), whereas ‘others’ defined cases expressing either both proteins at high levels, or both at low levels (KIF11^high^/KIF14^high^, KIF11^low^/KIF14^low^).

### 4.4. In Silico Analysis

Data including *KIF11* and *KIF14* expression levels of 277 CRC tumors and 303 noncancerous colon mucosa samples were downloaded from the TCGA and Genotype-Tissue Expression (GTEx) databases via UCSC Xena Browser (http://xena.ucsc.edu/, accessed on 22 June 2021) [65], whereas clinicopathological characteristics of CRC patients were obtained from the TCGA database using www.cBioPortal.org, accessed on 22 June 2021. *KIF11* and *KIF14* expression data were normalized by DESeq2 normalization and then divided into two groups based on the cut-off points determined with the Evaluate Cutpoints application [21]. Values lower than 11.81 for KIF11 and 9.63 for *KIF14* indicated negative expression of examined genes, while values equal or higher than established cut-off points were interpreted as positive. Cases with the coexpression of high levels of *KIF11* and *KIF14* were analyzed against those with the opposite expression profile (*KIF11*^low^/*KIF14*^low^), whereas “others” defined cases where only one gene was highly expressed (*KIF11*^high^/*KIF14*^low^, *KIF11*^low^/*KIF14*^high^). To identify prognostic factors for OS (median OS = 2475 days) in the TCGA cohort, a survival analysis based on data available for 271 CRC patients was performed. In addition, the top 50 genes positively correlated with *KIF11* or *KIF14* in colon adenocarcinoma were analyzed using the UALCAN web portal (http://ualcan.path.uab.edu/, accessed on 22 June 2021) [66] and TCGA dataset. Pathway analysis and visualization were performed using the Reactome pathway database (https://reactome.org, accessed on 22 June 2021) [67], while the Kyoto Encyclopedia of Genes and Genomes (KEGG) BRITE (https://www.genome.jp/kegg/brite.html, accessed on 22 June 2021) was used to examine functional hierarchies of *KIF11* or *KIF14* and the top 50 coexpressed genes. The STRING database (https://string-db.org, accessed on 30 August 2021) [68] and Cytoscape tool [69] were utilized to construct a protein-protein interaction network (PPI) of the top 50 genes coexpressed with either *KIF11* or *KIF14*. To find the Gene Ontology (GO) categories (cellular component (CC), biological process (BP) and molecular function (MF)) shared by *KIF11*- or *KIF14*-correlated genes, the Database for Annotation, Visualization and Integrated Discovery (DAVID; https://david.ncifcrf.gov, accessed on 30 August 2021) [70] was used.

### 4.5. Statistical Analysis

Statistical analysis was performed with GraphPad Prism (v8.0; GraphPad Software, San Diego, CA, USA) and SPSS software packages (v26.0, IBM Corporation, Armonk, NY, USA). Data normality was evaluated using the Shapiro Wilk test. Comparative analysis was carried out with the Mann-Whitney test for continuous variables and the Chi-squared or Fisher’s exact tests for categorical variables. The correlations between the expression of KIF11 and KIF14 were evaluated by utilizing the Spearman correlation coefficient. Survival curves were prepared with the Kaplan Meier estimation and compared by the Mantel-Cox log-rank test. To estimate the hazard ratios (HR) with 95% confidence intervals (CI), univariate and multivariate survival analyses were performed by the Cox proportional hazards model. The variables with amounts of missing data over 20% (PNI, VI) were not considered for the model. Details on missing data are depicted in Supplementary Table S2. For the in-house cohort, adjustment variables included age at diagnosis (continuous variable), gender (male vs. female), tumor grade (intermediate vs. high), pT (T1-T2 vs. T3-T4), pN (N0 vs. N1-N2), pM (absent vs. present), and the study. For multivariate survival analysis of in silico data, covariates were age at diagnosis (continuous variable), gender (male vs. female), tumor grade (low and intermediate vs. high), and stage (I-II vs. III-IV). Due to the strong correlation noted between *KIF11* and *KIF14* mRNA expression levels, two separate multivariate models for these transcripts were built. A *p* value < 0.05 was considered statistically significant.

## 5. Conclusions

In conclusion, our results suggest that CRC patients can be stratified into distinct risk categories not only by established clinicopathological factors but also by biological and molecular determinants such as KIF11 and KIF14 expression. Mechanistically, this is likely attributable to their role in maintaining genome integrity.

## Figures and Tables

**Table 1:** Association of KIF11 and KIF14 protein expression in colorectal cancer with patient characteristics.

Clinicopathological Feature	n (%) n = 86	KIF11 Expression		*p* Value	KIF14 Expression		*p* Value
		Low n = 61	High n = 25		Low n = 57	High n = 29	
Age (years)							
≤65	38 (44.19)	29 (76.32)	9 (23.68)	0.35	27 (71.05)	11 (28.95)	0.49
>65	48 (55.81)	32 (66.67)	16 (33.33)		30 (62.50)	18 (37.50)	
Gender							
Male	49 (56.98)	33 (67.35)	16 (32.65)	0.48	35 (71.43)	14 (28.57)	0.26
Female	37 (43.02)	28 (75.68)	9 (24.32)		22 (59.46)	15 (40.54)	
Grading							
G2	76 (91.57)	54 (71.05)	22 (28.95)	>0.99	50 (65.79)	26 (34.21)	>0.99
G3	7 (8.43)	5 (71.43)	2 (28.57)		5 (71.43)	2 (28.57)	
pT status							
T2	13 (15.12)	10 (76.92)	3 (23.08)	0.67	9 (69.23)	4 (30.77)	0.68
T3	60 (69.77)	42 (70.00)	18 (30.00)		38 (63.33)	22 (36.67)	
T4	13 (15.12)	9 (69.23)	4 (30.77)		10 (76.92)	3 (23.08)	
pN status							
N0	33 (40.74)	25 (75.76)	8 (24.24)	0.62	20 (60.61)	13 (39.39)	0.48
N1-N2	48 (59.26)	33 (68.75)	15 (31.25)		33 (68.75)	15 (31.25)	
pM status							
M0	42 (52.50)	29 (69.05)	13 (30.95)	>0.99	27 (64.29)	15 (35.71)	>0.99
M1	38 (47.50)	27 (71.05)	11 (28.95)		25 (65.79)	13 (34.21)	
VI							
Absent	24 (60.00)	16 (66.67)	8 (33.33)	0.53	9 (37.50)	15 (62.50)	**0.01**
Present	16 (40.00)	9 (56.25)	7 (43.75)		13 (81.25)	3 (18.75)	
PNI							
Absent	25 (89.29)	14 (56.00)	11 (44.00)	0.26	12 (48.00)	13 (52.00)	>0.99
Present	3 (10.71)	3 (100.00)	0 (0.00)		2 (66.67)	1 (33.33)	

Abbreviations: VI—vascular invasion, PNI—perineural invasion. Significant *p*-value (*p* < 0.05) is marked in bold.

**Table 2:** Univariate and multivariate Cox proportional hazards models for OS of our cohort of CRC patients.

Variable	Univariate Cox				Multivariate Cox: KIF11(PS) and KIF14				Multivariate Cox: KIF11(IRS)/KIF14				Multivariate Cox: KIF11(PS)/KIF14			
	HR	95%CI		*p*	HR	95%CI		*p*	HR	95%CI		*p*	HR	95%CI		*p*
		Lower	Upper			Lower	Upper			lower	upper			Lower	Upper	
KIF11 (IRS)	1.51	0.79	2.87	0.21	-	-	-	-	-	-	-	-	-	-	-	-
KIF11 (PS)	2.17	1.14	4.12	**0.02**	2.41	1.10	5.28	**0.03**	-	-	-	-	-	-	-	-
KIF14	0.54	0.26	1.10	0.09	0.43	0.17	1.11	0.08	-	-	-	-	-	-	-	-
KIF11(IRS)^low^/KIF14^high^	Ref.				-	-	-	-	Ref.				-	-	-	-
KIF11(IRS)^high^/KIF14^low^	3.33	1.29	8.55	**0.01**	-	-	-	-	3.91	1.13	13.54	**0.03**	-	-	-	-
Others	0.91	0.39	2.15	0.84	-	-	-	-	1.28	0.43	3.82	0.66	-	-	-	-
KIF11(PS)^low^/KIF14^high^	Ref.				-	-	-	-	-	-	-	-	Ref.			
KIF11(PS)^high^/KIF14^low^	3.29	1.29	8.37	**0.01**	-	-	-	-	-	-	-	-	5.72	1.74	18.83	**0.004**
Others	2.18	0.92	5.13	0.08	-	-	-	-	-	-	-	-	2.58	0.92	7.19	0.07
age	1.00	0.97	1.03	0.83	1.02	0.99	1.06	0.18	1.01	0.98	1.05	0.48	1.02	0.99	1.06	0.17
gender	1.06	0.57	1.99	0.85	1.85	0.84	4.06	0.13	1.81	0.77	4.23	0.17	1.86	0.85	4.08	0.12
grade	2.31	0.80	6.64	0.12	3.62	1.14	11.53	**0.03**	3.15	0.99	9.99	0.05	3.68	1.15	11.76	**0.03**
pT	2.14	0.84	5.47	0.11	0.83	0.26	2.65	0.75	0.95	0.30	3.02	0.92	0.85	0.27	2.70	0.78
pN	1.77	0.90	3.48	0.10	1.57	0.70	3.54	0.28	1.16	0.47	2.84	0.75	1.58	0.70	3.55	0.27
pM	3.27	1.64	6.53	**0.001**	3.09	1.37	6.99	**0.007**	2.80	1.22	6.40	**0.02**	3.03	1.35	6.80	**0.007**

Abbreviations: CI—confidence interval, CRC—colorectal cancer, HR—hazard ratio, IRS—immunoreactive score, OS—overall survival, PS—immunopercentage. “-” indicates variable was not included in multivariate Cox analysis. Significant *p*-values (*p* < 0.05) are indicated in bold.

**Table 3:** Association of *KIF11* and *KIF14* transcript expression in colorectal cancer with patient characteristics.

Clinicopathological Feature	n (%) n = 277	*KIF11* Expression		*p* Value	*KIF14* Expression		*p* Value
		Low n = 244	High n = 33		Low n = 194	High n = 83	
Age (years)							
≤65	129 (46.91)	114 (88.37)	15 (11.63)	>0.99	91 (70.54)	38 (29.46)	>0.99
>65	146 (53.09)	128 (87.67)	18 (12.33)		102 (69.86)	44 (30.14)	
Gender							
Male	150 (54.55)	136 (90.67)	14 (9.33)	0.14	103 (68.67)	47 (31.33)	0.60
Female	125 (45.45)	106 (84.80)	19 (15.20)		90 (72.00)	35 (28.00)	
pT status							
T1	6 (2.18)	6 (100.00)	0 (0.00)	0.36	4 (66.67)	2 (33.33)	0.34
T2	43 (15.64)	38 (88.37)	5 (11.63)		34 (79.07)	9 (20.93)	
T3	188 (68.36)	166 (88.30)	22 (11.70)		132 (70.21)	56 (29.79)	
T4	38 (13.82)	32 (84.21)	6 (15.79)		23 (60.53)	15 (39.47)	
pN status							
N0	160 (58.18)	143 (89.38)	17 (10.63)	0.45	120 (75.00)	40 (25.00)	**0.045**
N1-N2	115 (41.82)	99 (86.09)	16 (13.91)		73 (63.48)	42 (36.52)	
pM status							
M0	185 (83.33)	162 (87.57)	23 (12.43)	0.27	130 (70.27)	55 (29.73)	0.84
M1	37 (16.67)	35 (94.59)	2 (5.41)		25 (67.57)	12 (32.43)	
TNM stage							
I-II	150 (55.97)	133 (88.67)	17 (11.33)	0.58	115 (76.67)	35 (23.33)	**0.01**
III-IV	118 (44.03)	102 (86.44)	16 (13.56)		73 (61.86)	45 (38.14)	

Significant *p*-values (*p* < 0.05) are marked in bold.

**Table 4:** Univariate and multivariate Cox proportional hazards models for OS of TCGA cohort of CRC patients.

Variable	Univariate Analysis				Multivariate Analysis: *KIF11*				Multivariate Analysis: *KIF14*				Multivariate Analysis: *KIF11*/*KIF14*			
	HR	95% CI		*p*	HR	95%CI		*p*	HR	95%CI		*p*	HR	95%CI		*p*
		Lower	Upper			Lower	Upper			Lower	Upper			Lower	Upper	
*KIF11*	0.36	0.13	0.996	**0.049**	0.32	0.11	0.89	**0.03**	-	-	-	-	-	-	-	-
*KIF14*	0.66	0.38	1.17	0.16	-	-	-	-	0.47	0.26	0.86	**0.02**	-	-	-	-
*KIF11*^low^/*KIF14*^low^	Ref.				-	-	-	-	-	-	-	-	Ref.			
*KIF11*^high^/*KIF14*^high^	0.27	0.08	0.88	**0.03**	-	-	-	-	-	-	-	-	0.22	0.07	0.71	**0.01**
Others	1.10	0.60	1.99	0.77	-	-	-	-	-	-	-	-	0.74	0.39	1.41	0.36
age	1.02	0.998	1.04	**0.08**	1.03	1.004	1.05	**0.02**	1.03	1.01	1.05	**0.004**	1.03	1.01	1.05	**0.01**
gender	1.43	0.87	2.35	0.16	1.30	0.76	2.20	0.34	1.38	0.81	2.35	0.24	1.36	0.80	2.33	0.26
pT	3.21	1.17	8.84	**0.02**	-	-	-	-	-	-	-	-	-	-	-	-
pN	2.46	1.50	4.04	**<0.0001**	-	-	-	-	-	-	-	-	-	-	-	-
pM	4.21	2.34	7.56	**<0.0001**	-	-	-	-	-	-	-	-	-	-	-	-
stage	2.60	1.55	4.35	**<0.0001**	3.37	1.96	5.77	**<0.0001**	3.83	2.18	6.73	**<0.0001**	3.65	2.08	6.41	**<0.0001**

Abbreviations: CI—confidence interval, CRC—colorectal cancer, HR—hazard ratio, OS—overall survival. “-” indicates variable was not included in multivariate Cox analysis. Significant *p*-values (*p* < 0.05) are indicated in bold.

## Data Availability

Publicly available datasets were analyzed in this study. These data can be found here: http://www.cbioportal.org/study/summary?id=paad_tcga_pan_can_atlas_2018 (accessed on 25 May 2021); https://xenabrowser.net (accessed on 25 May 2021), http://ualcan.path.uab.edu/ (accessed on 22 June 2021); https://string-db.org, accessed on 30 August 2021; https://david.ncifcrf.gov, accessed on 30 August 2021. Our own data presented in this study are available on request from the corresponding author. The data are not publicly available due to ethical restrictions.

## Supplementary Materials

The following are available online at https://www.mdpi.com/article/10.3390/ijms22189732/s1.
