# Supplementary material for: Prognostic Impact and Functional Annotations of KIF11 and KIF14 Expression in Patients with Colorectal Cancer
Source: Int J Mol Sci. 2021 Sep 8;22(18):9732. doi: 10.3390/ijms22189732 (PMC8466126; doi:10.3390/ijms22189732)
Supplement: Supplementary file 1 [file ijms-22-09732-s001.zip › Supplementary file.pdf]

Table S1. Association of KIF11 protein expression (PS score) in colorectal cancer with patient characteristics.

Clinicopathological feature	n (%) n = 86	KIF11 (PS) expression		*p* value
		negative n = 63	positive n = 23	
Age (years)				
≤ 65	38 (44.19)	29 (76.32)	9 (23.68)	0.63
> 65	48 (55.81)	34 (70.83)	14 (29.17)	
Gender				
Male	49 (56.98)	36 (73.47)	13 (26.53)	>0.99
Female	37 (43.02)	27 (72.97)	10 (27.03)	
Grading				
G2	76 (91.57)	57 (75.00)	19 (25.00)	>0.99
G3	7 (8.43)	5 (71.43)	2 (28.57)	
pT status				
T2	13 (15.12)	9 (69.23)	4 (30.77)	
T3	60 (69.77)	48 (80.00)	12 (20.00)	0.18
T4	13 (15.12)	6 (46.15)	7 (53.85)	
pN status				
N0	33 (40.74)	23 (69.70)	10 (30.30)	0.43
N1-N2	48 (59.26)	38 (79.17)	10 (20.83)	
pM				
M0	42 (52.50)	32 (76.19)	10 (23.81)	0.62
M1	38 (47.50)	27 (71.05)	11 (28.95)	
VI				
Absent	24 (60.00)	20 (83.33)	4 (16.67)	0.69
Present	16 (40.00)	12 (75.00)	4 (25.00)	
PNI				
Absent	25 (89.29)	20 (80.00)	5 (20.00)	>0.99
Present	3 (10.71)	3 (100.00)	0 (0.00)	

Abbreviations: VI - vascular invasion, PNI - perineural invasion

Table S2. Missing data on clinicopathological features of CRC patients.

Variable	Missing data of our cohort (%)	Missing data of TCGA cohort (%)
survival time	8 (10.7)	4 (1.5)
survival status	0 (0)	4 (1.5)
grade	2 (2.7)	-
pN	3 (4.0)	0 (0)
pM	6 (8.0)	53 (19.3)
stage	-	7 (2.5)
VI	40 (53.3)	-
PNI	50 (66.7)	-

Abbreviations: CRC – colorectal cancer, VI - vascular invasion, PNI - perineural invasion, TCGA - The Cancer Genome Atlas. '-' indicates a lack of data on variables.

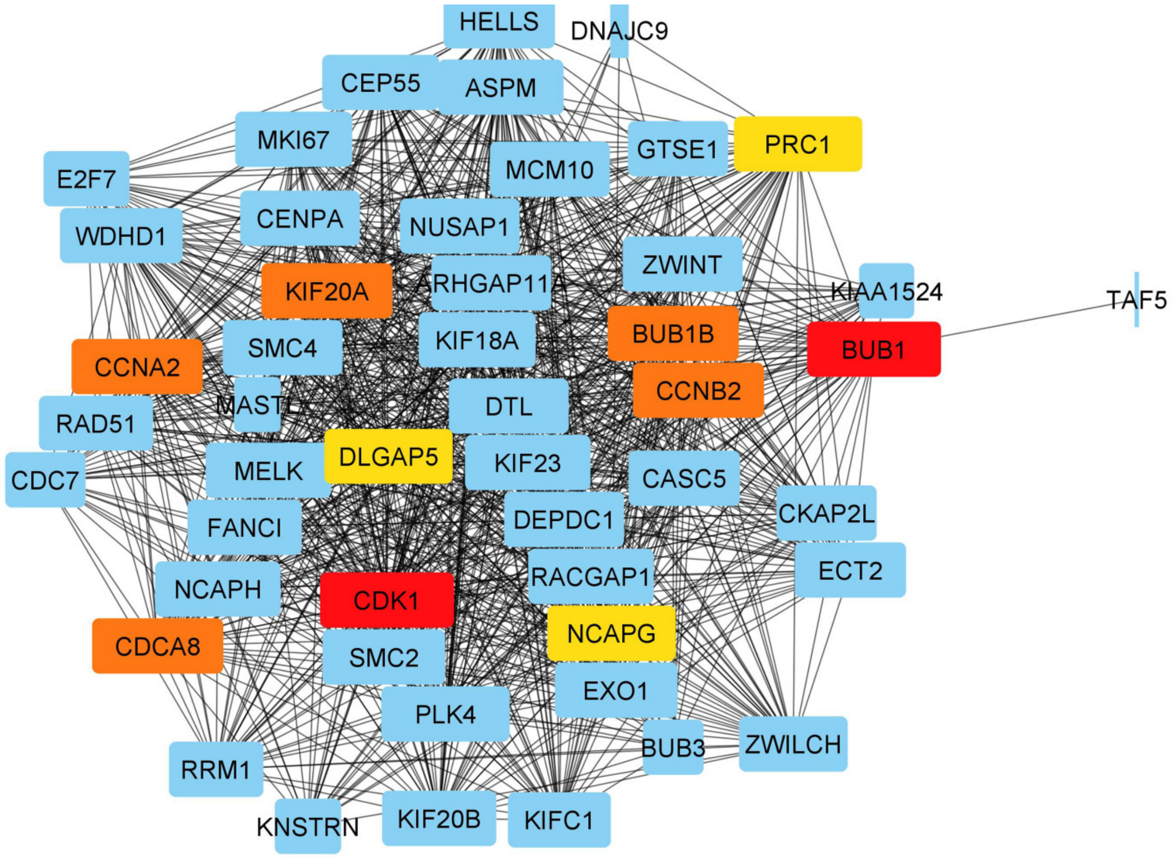

Figure S1. Protein-protein interaction network for the top 50 genes positively correlated with *KIF11*. In the visualized network, the degrees of connections of the nodes are symbolized by their areas. Top 10 hub genes in the *KIF11*-correlated network identified by CytoHubba Cytoscape plugin are highlighted in a red to yellow gradient. The deeper color the higher degree of enrichment. Other nodes in the network are highlighted in blue.

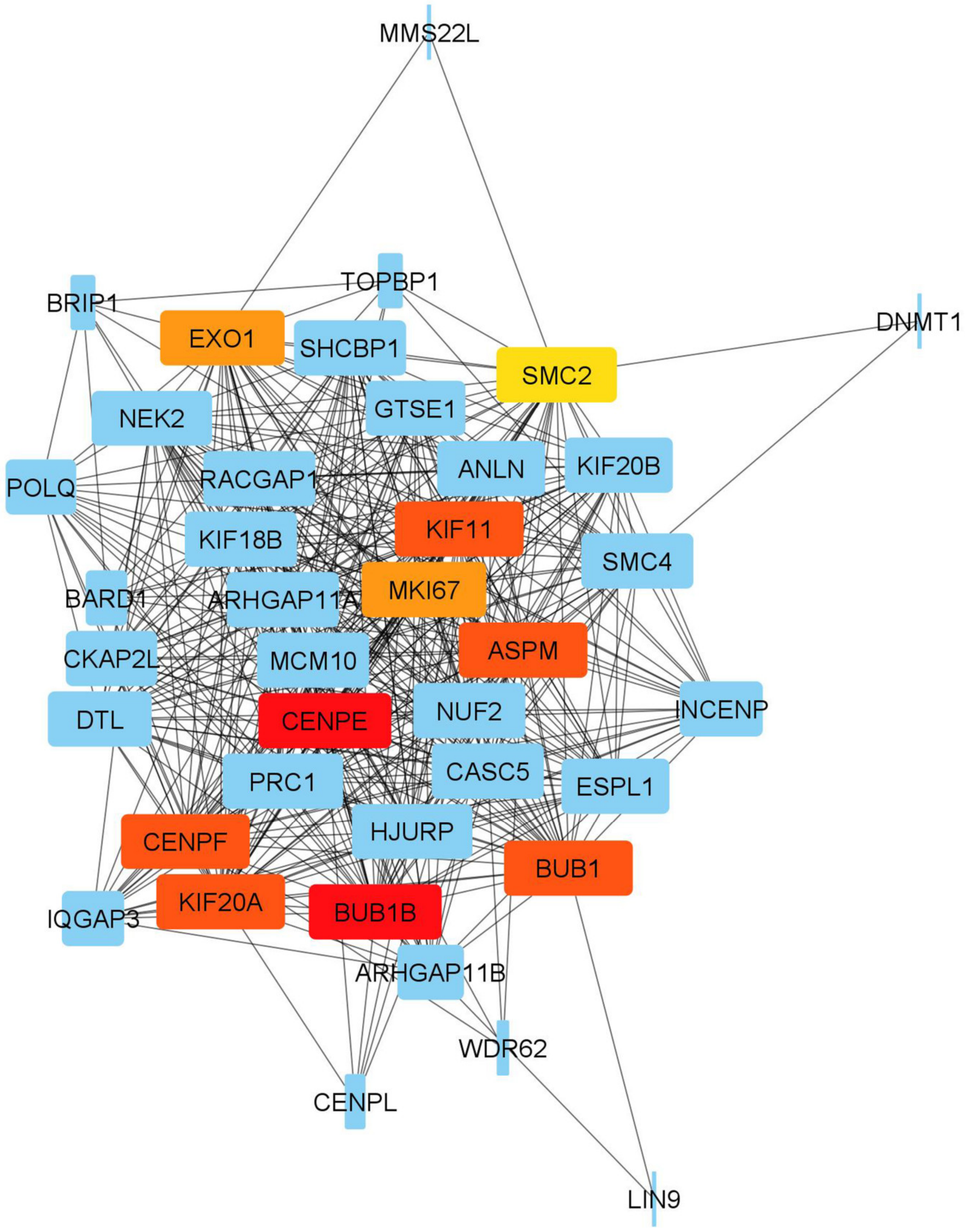

Figure S2. Protein-protein interaction network for the top 50 genes positively correlated with *KIF14*. In the visualized network, the degrees of connections of the nodes are symbolized by their areas. Top 10 hub genes in the *KIF14*-correlated network identified by CytoHubba Cytoscape plugin are highlighted in a red to yellow gradient. The deeper color the higher degree of enrichment. Other nodes in the network are highlighted in blue. Disconnected nodes were hidden.
