# Supplementary figures and images for: Prognostic Impact and Functional Annotations of KIF11 and KIF14 Expression in Patients with Colorectal Cancer
Source: Int J Mol Sci. 2021 Sep 8;22(18):9732. doi: 10.3390/ijms22189732 (PMC8466126; doi:10.3390/ijms22189732)

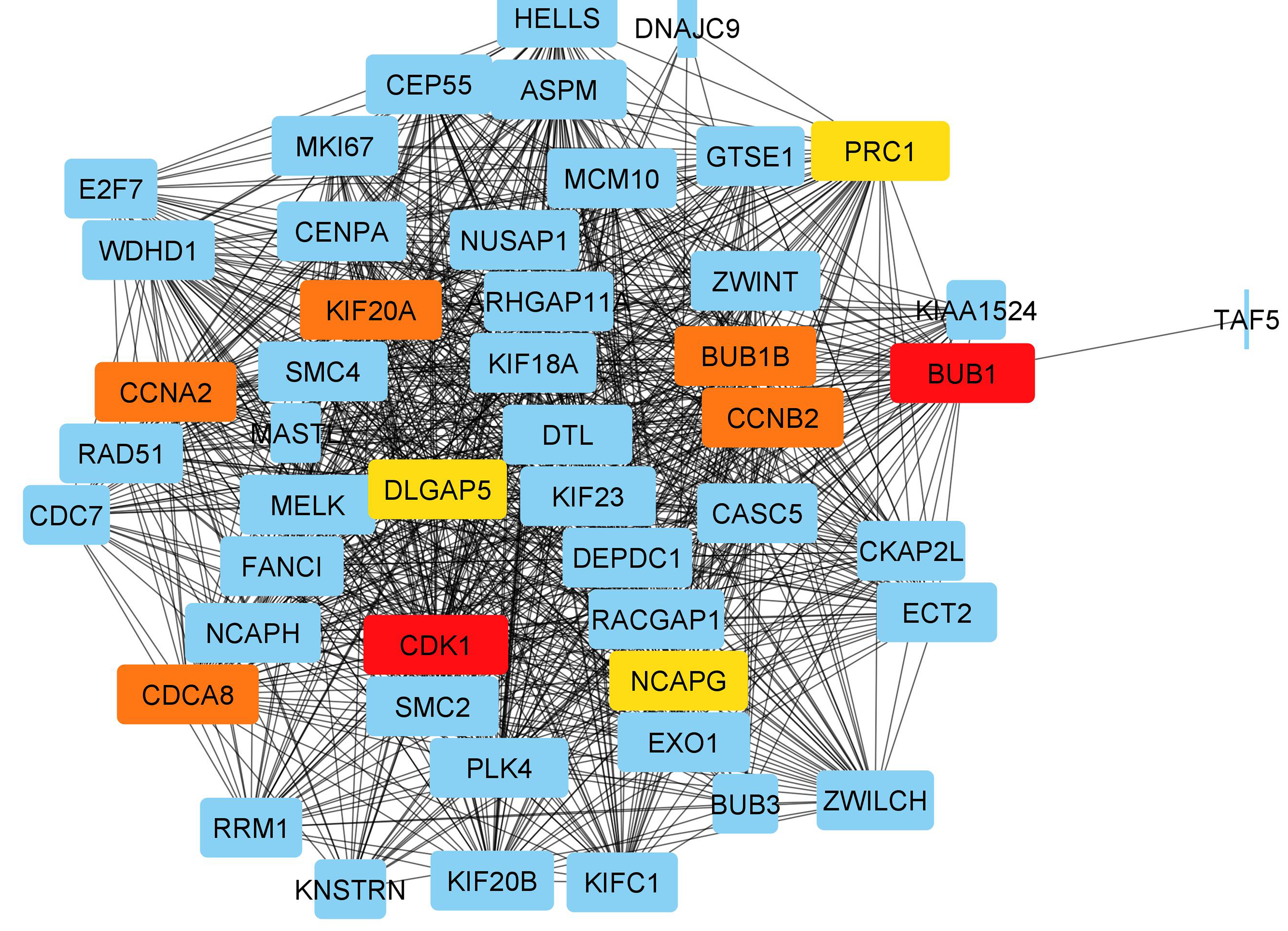

Supplement: Supplementary file 1 [file ijms-22-09732-s001.zip › Figure S1.tif]

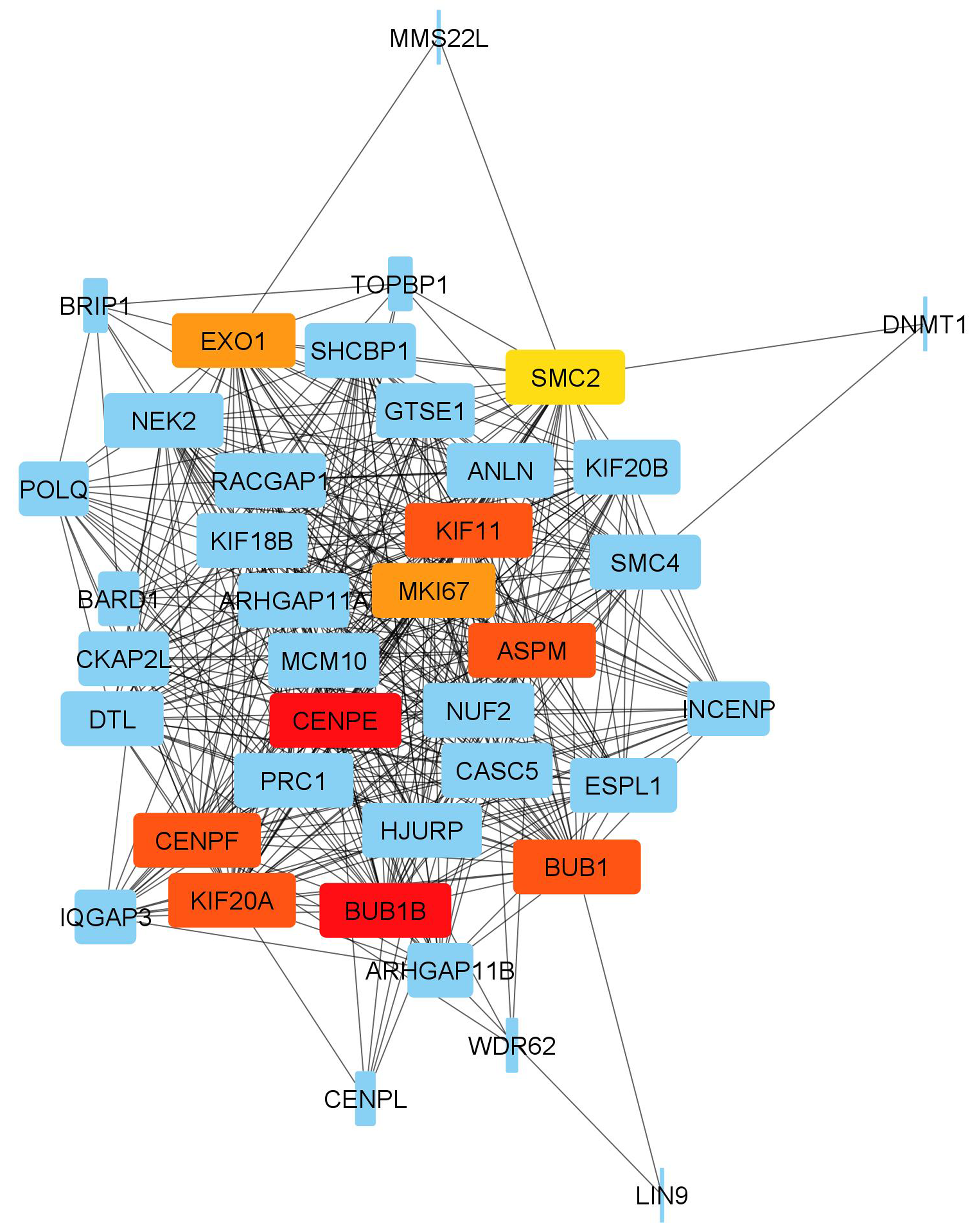

Supplement: Supplementary file 1 [file ijms-22-09732-s001.zip › Figure S2.tif]
